# Comment on “Post‐Botulinum Headache in Cosmetic Practice: A Prospective Study”

**DOI:** 10.1111/jocd.70810

**Published:** 2026-03-23

**Authors:** Neeraj Singh, Monika Srivastav

**Affiliations:** ^1^ Dr. D. Y. Patil Medical College Hospital and Research Centre Dr. D. Y. Patil Vidyapeeth (Deemed‐to‐be‐University) Pune India; ^2^ Dr. D. Y. Patil Dental College and Hospital Dr. D. Y. Patil Vidyapeeth (Deemed‐to‐be‐University) Pune India

**Keywords:** abobotulinumtoxinA, adverse events, cosmetic dermatology, headache phenotyping, post‐botulinum headache

To the Editor,

We read with great interest the prospective study by Akpınar and colleagues evaluating the incidence and clinical profile of headache following cosmetic abobotulinumtoxinA injections [1]. The structured follow up at multiple time points and the inclusion of both facial and nonfacial treatment sites provide useful real world granularity. The authors also offer clinically relevant descriptive data regarding headache timing and symptom burden. However, several interpretive and methodological considerations merit closer examination.

An important issue relates to the binary modeling of headache occurrence without accounting for exposure intensity across anatomical regions. Although all headache cases occurred after upper facial injections, the analysis does not appear to adjust for the markedly higher proportion of patients receiving treatment in these trigeminally innervated areas. Because 92% of the cohort underwent upper facial injections, the observed site specific association may partly reflect exposure density rather than true regional susceptibility [2]. This distinction matters for procedural counseling, since clinicians may otherwise overattribute risk to facial anatomy. Region adjusted modeling or multivariable logistic regression incorporating injection distribution could better clarify whether upper facial treatment independently predicts headache.

Interpretation of the first time recipient effect also warrants deeper analytical framing. The reported difference of 45% versus 8.8% suggests a strong exposure gradient, yet the analysis relies primarily on unadjusted categorical comparisons. Without modeling potential covariate interactions such as age, migraine history, or cumulative toxin exposure, the magnitude of the association may be directionally inflated. For clinical decision making, distinguishing whether this represents a true biologic sensitization phenomenon or residual confounding is important when counseling new aesthetic patients. A propensity adjusted or multivariable framework would strengthen causal inference and improve risk communication.

The study's descriptive characterization of headache phenotype is informative but stops short of integrating standardized headache classification constructs. Pain types were recorded using heterogeneous descriptors such as pressure type and tension type, which may overlap within International Classification of Headache Disorders based phenotypes [3]. Greater phenotypic harmonization could help determine whether post injection symptoms cluster toward tension type headache, procedural nociceptive pain, or migraine‐like activation. This refinement has practical implications because management strategies and patient reassurance differ across headache subtypes [4]. Future work incorporating structured diagnostic criteria and patient‐reported outcome measures would enhance clinical interpretability.

Overall, this prospective analysis provides valuable early insight into post procedural headache patterns in cosmetic practice. We commend the authors for addressing an underexplored yet clinically relevant adverse effect and for implementing systematic follow up. Further analytically adjusted and phenotype refined studies may help delineate true risk drivers and support more individualized patient counseling in aesthetic neurotoxin practice.

## Author Contributions

**Neeraj Singh:** conceptualization, methodology, validation, supervision, project administration, writing – original draft, writing – review and editing. **Monika Srivastav:** writing – original draft, writing – review and editing.

## Funding

The authors have nothing to report.

## Ethics Statement

The authors have nothing to report.

## Conflicts of Interest

The authors declare no conflicts of interest.

## Data Availability

The authors have nothing to report.
